# Rate of fractional change in corneal tomography parameters in keratoconus using a combination of predictive parameters

**DOI:** 10.1186/s40662-023-00357-y

**Published:** 2023-10-01

**Authors:** Gaurav Prakash, Alex Mammen, Vishal Jhanji

**Affiliations:** 1 Department of Ophthalmology, University of Pittsburgh School of Medicine, Pittsburgh, PA USA; 2 University of Pittsburgh Medical Center, 203 Lothrop St, Suite 800, Pittsburgh, PA 15213 USA

**Keywords:** Keratoconus, Progression, Corneal tomography, Predictive parameters

## Abstract

**Background:**

To compare the rate of fractional change for multiple corneal tomographic factors in progressive keratoconus (KC).

**Methods:**

In this retrospective case series, 40 eyes (40 patients) with progressive KC (increase in central keratometry of 1.00 D or maximum keratometry of 1.50 D on two visits at least six months apart) were included. Cases with previous history of ocular surgery, poor scans, corneal scars, severe dry eyes, post-excimer ectasia, pellucid marginal degeneration were excluded. Medical records, corneal tomography and anterior corneal wavefront (8 mm) (Scheimpflug tomography, Pentacam, Oculus, Germany) were analyzed. Rate of fractional change (Rx = (x_1_ − x_0_)/(|x_0_|t_m_)); where, x_1_ = value at follow-up, x_0_ = value at initial visit and t_m_ = time in months, was measured.

**Results:**

The mean age of the patients was 30.0 ± 8.4 years. The mean follow-up duration was 8.9 ± 4.2 months. Coma (0.076 ± 1.4) had the largest rate of fractional change (*P* = 1.7 × 10^−14^, Kruskal–Wallis test). The rate of fractional change was higher for aberrometric parameters (anterior corneal higher-order aberrations root mean square and anterior coma) compared to pachymetric and keratometric parameters (*P* values ranging from 1.4 × 10^−4^ to 7.4 × 10^−10^, Mann–Whitney test, effect size ranging from 0.4–0.7). The rate of fractional change was comparable between pachymetric and keratometric factors (*P* > 0.05 for all comparisons, Mann–Whitney test).

**Conclusions:**

Anterior corneal wavefront, especially anterior coma, were noted to have higher rate of fractional change compared to single point keratometric and pachymetric indices in progressive KC. This information can be used for decision-making when monitoring patients with KC.

**Supplementary Information:**

The online version contains supplementary material available at 10.1186/s40662-023-00357-y.

## Background

Keratoconus (KC) is a progressive corneal disorder associated with ectasia and thinning [1, 2]. Multiple classification systems have attempted to define progressive KC, typically based on an increase in keratometry with or without a decrease in pachymetry and visual acuity during longitudinal follow-up [3–8]. Although an association between predictive factors for KC severity has been previously demonstrated, the relationship is non-linear [9].

Most biological non-linear predictive models result from one component changing faster than the other over the same time, and these different slopes when compared to time can be viewed as non-linear constructs when these variables are themselves compared. This is distinct from linear predictive models, where both components tend to maintain the same slope in relation to time. Hence, there is potential to investigate if these predictive factors worsen at different rates over a given period. If so, it may be possible to expedite the decision for therapeutic interventions, such as cross-linking, to preserve visual acuity and prevent irreversible progression.

The aim of this study was to compare the rate of change in pachymetric, keratometric, and corneal wavefront-related parameters over a standardized change in time and base values (defined as the fractional rate of change later) in cases with progressive KC.

## Methods

This retrospective study was conducted at a tertiary care hospital. The study protocol was approved by the Institutional Review Board of the University of Pittsburgh (Protocol 19010171), and it adhered to the principles outlined in the Declaration of Helsinki. Clinical data were obtained by reviewing case records. Corneal tomography and wavefront data were acquired using Scheimpflug tomography (Pentacam, Oculus, Wetzlar, Germany). The criteria for disease progression were defined as an increase in central keratometry of 1.00 D or maximum keratometry (Kmax) of 1.50 D in at least two visits, with a minimum interval of six months between visits [3]. Cases with a history of previous ocular surgery, including corneal cross-linking, poor-quality scans, corneal scars, severe dry eyes, post-excimer ectasia, and pellucid marginal degeneration, were excluded.

### Statistical analysis

Tomographic data was extracted from the device's csv files (load files, Pentacam, Oculus, Wetzlar, Germany). Corneal wavefront data was computed at a radius of 8 mm. The data were entered into an Excel worksheet (Microsoft, Richmond, VA) and subsequently analyzed using SPSS 16.0 (SPSS Inc., Illinois).

The fractional change is defined as the difference in the parameter being evaluated [e.g., Kmax, central corneal thickness (CCT)] between the follow-up visit and the initial visit, divided by the absolute value (modulus) at the initial visit and the time elapsed in months.

The rate of fractional change (Rx) was mathematically expressed as:

$$\mathrm{Rx}= ({\mathrm{x}}_{1}- {\mathrm{x}}_{0}) / (|{\mathrm{x}}_{0}|{\mathrm{t}}_{\mathrm{m}})$$

where, x_1_ = value at follow-up visit; x_0_ = value at initial visit; |x_0_|= modulus of value at initial visit; t_m_ = time in months (days between visits/30).

In terms of intuition, this fractional rate of change is the change occurring per unit change in the parameter as well as per unit change in time, thus creating a mathematically level playing field for comparison. Using the absolute value for the denominator ensured that the directionality of the change is not lost due to algebraic reduction. Furthermore, when asymmetric aberrations were involved, laterality (right or left) was considered in all calculations.

The concept of fractional change is derived from the concept of creating a normalized magnification scale. For example, a change of 1 unit with a base denominator of 10 is not the same as the change of 2 units with a based denominator of 50. Comparing an actual change normalized to the base value results in a fair comparison for the parameters being evaluated. The result is a ‘fraction’ and hence the fractional rate of change when this parameter is divided by the amount of time elapsed.

The distribution of the Rx was found to be non-normal (Kolmogorov–Smirnov test, *P* < 0.05 for all parameters). Therefore, nonparametric tests were employed. Descriptive data were presented using measures of central tendency in the format of mean ± standard deviation and median values. The difference in means between observations was assessed using the Kruskal–Wallis test. Nonparametric post hoc analysis was conducted using the Rank Sum test. The effect size for the nonparametric test was calculated using the formula:

$$\mathrm{S}=\mathrm{z} / (\mathrm{n}^{0.5})$$

where S represents the effect size, z is the Z-score from the Rank Sum test, and n is the total sample size. An effect size of 0.5 was considered large, 0.3 was considered medium, and 0.1 was considered small [10, 11].

A-priori sample size estimation: Pilot data for 10 cases was used to calculate the estimated sample size for an alpha of 0.05, beta of 0.2 (power of 0.8), the standard deviation of change as 4.3 and r within as 0.97. The estimated minimal sample size was 31 cases. We included 40 cases to ensure sufficient post hoc power.

The analysis was conducted in two steps:

Step 1: Pachymetric, keratometric, and 8 mm anterior corneal higher-order aberrations root mean square (HOARMS) related variables were compared to select the ones exhibiting growth rates.

Step 2: The selected variables were compared between different groups to assess those with the greatest rate of fractional change.

Furthermore, the rate of fractional change was compared between two age groups (≤ 30 years and > 30 years) and between genders to evaluate the impact of age on progression.

## Results

A total of 40 eyes of 40 KC patients were included in the analysis. Per Amsler-Krumeich Classification of KC, the severity distribution was Grade 1: 15, Grade 2: 4, Grade 3: 4, Grade 4:17 [12]. The mean age was 30.0 ± 8.4 years (range 17–43 years). There were 17 females. The mean follow-up duration was 8.9 ± 4.2 months (range 6 to 24 months). All the included parameters noted change in mean values in trend with KC progression (Table 1). The rates of fractional change are noted in Table 1.

**Table 1:** Difference in value and rate of fractional change in parameters in patients with progressive keratoconus

Parameter		Kf (D)	Ks (D)	Kmax (D)	CA-HOA (µm)	Coma (µm)	SA (µm)	TCT (µm)	ACT (µm)	CCT (µm)	CV7 (mm^3^)	CV10 (mm^3^)
Initial visit (x_0_)	Mean	51.8	56.7	66.6	8.7	6.1	− 3.5	429.4	446.8	465.6	23.0	57.4
	SD	9.4	10.6	13.5	4.1	3.6	3.9	76.2	72.7	68.4	1.8	4.7
	Median	51.2	56.8	69.1	8.8	5.6	− 3.0	424.5	435	464	22.5	56.7
Follow-up visit (x_1_)	Mean	53.2	58.8	70.6	9.8	6.8	− 4.2	406.8	426.0	450.4	22.7	56.9
	SD	10.2	11.1	14.9	5.0	3.7	4.2	71.5	69.9	65.9	1.9	4.9
	Median	52.9	58.6	71	10.2	7.2	− 3.6	400	421	447.5	22.8	57.2
Rate of fractional change*	Mean	0.003	0.005	0.007	0.034	0.076	− 0.024	− 0.007	− 0.007	− 0.005	− 0.002	− 0.001
	SD	0.009	0.008	0.010	0.105	0.140	0.193	0.009	0.010	0.009	0.009	0.010

*Kf* = flat central keratometry (anterior); *Ks* = steep central keratometry (anterior); *Kmax* = maximum keratometry (anterior); *CA-HOA* = corneal anterior surface higher-order aberrations at 8 mm; *SA* = spherical aberration, anterior surface at 8 mm; *Coma* = coma, anterior surface at 8 mm; *TCT* = thinnest corneal thickness; *ACT* = apical corneal thickness; *CCT* = central corneal thickness; *CV7* = corneal volume at 7 mm; *CV10* = corneal volume at 10 mm; *SD* = standard deviation

^*^Rate of fractional change = (x_1_ − x_0_)/(|x_0_|t_m_), where t_m_ is time in months

### Comparison between rate of fractional change

#### Step 1: Intragroup analysis

Keratometry: The following anterior corneal parameters were included: Kmax, steep central keratometry, and flat central keratometry (Kf). Significant differences were noted in the rate of fractional change in keratometric factors (Kruskal–Wallis test, *P* = 1.6 × 10^−6^). Both Kmax and steep central keratometry front had significantly greater progression rates compared to flat central keratometry (*P* = 2.3 × 10^−6^, effect size = 0.5, and *P* = 2.2 × 10^−4^, effect size = 0.4 respectively, Mann–Whitney test). Kmax and Ks did not exhibit a significant difference (*P* = 0.1, Mann–Whitney test) in progression rates.

Pachymetry: Thinnest corneal thickness (TCT), apical corneal thickness (ACT), pupil-centric CCT, corneal volume at 7 mm (CV7) and corneal volume at 10 mm (CV10) were compared. The rate of fractional change for all corneal thickness parameters was significantly higher than the volumetric parameters (Kruskal–Wallis test, *P* = 3.2 × 10^−7^). Intra-group analysis suggested effect sizes ranging from 0.3 to 0.5 for pachymetric and volumetric factors (Table 2). Three corneal thickness parameters (TCT, ACT and CCT) had comparable rate of fractional change (Kruskal Wallis test, *P* = 0.6).

**Table 2:** Comparison between keratometry, pachymetry and higher-order aberrations in patients with progressive keratoconus

Comparison of pachymetric parameters^#^				
	ACT	CCT	CV7	CV10
TCT	*P* = 0.7, S = 0	*P* = 0.4, S = 0.1	***P* = 1.4 × 10**^**−4**^**, S = 0.4**	***P* = 1.7 × 10**^**−5**^**, S = 0.5**
ACT		*P* = 0.5, S = 0.1	***P* = 7.5 × 10**^**−4**^**, S = 0.4**	***P* = 2.3 × 10**^**−5**^**, S = 0.5**
CCT			***P* = 2.6 × 10**^**−3**^**, S = 0.3**	***P* = 1.6 × 10**^**−5**^**, S = 0.4**
CV10				*P* = 0.3, S = 0.1

Comparison of best performing keratometric, pachymetric and corneal higher order aberrations parameters^#^					
	CA-HOA	Coma	TCT	ACT	CCT
Ks	***P* = 7.3 × 10**^**−6**^**, S = 0.5**	***P* = 7.4 × 10**^**−10**^**, S = 0.7**	*P* = 0.1, S = 0.2	*P* = 0.3, S = 0.1	*P* = 0.6, S = 0.1
Kmax	***P* = 1.4 × 10**^**−4**^**, S = 0.4**	***P* = 1.9 × 10**^**−8**^**, S = 0.6**	*P* = 0.5, S = 0.1	*P* = 0.4, S = 0.1	*P* = 0.08, S = 0.2
CA-HOA			***P* = 7.3 × 10**^**−5**^**, S = 0.4**	***P* = 2.4 × 10**^**−4**^**, S = 0.4**	***P* = 2.9 × 10**^**−5**^**, S = 0.5**
Coma			***P* = 1.4 × 10**^**−8**^**, S = 0.6**	***P* = 3.4 × 10**^**−8**^**, S = 0.6**	***P* = 1.7 × 10**^**−9**^**, S = 0.7**

*Kmax* = maximum keratometry (anterior); *Ks* = steep central keratometry (anterior); *ACT* = apical corneal thickness; *CCT* = central corneal thickness; *TCT* = thinnest central cornea; *CV7* = corneal volume at 7 mm; *CV10* = corneal volume at 10 mm; *CA-HOA* = corneal anterior surface higher-order aberrations at 8 mm; *Coma* = coma, anterior surface at 8 mm

^#^*P* value and strength of effect (S), Mann–Whitney test. Significant *P* values < 0.05 and S ≥ 0.3 are in bold

Corneal anterior HOARMS: Corneal front surface HOARMS (CA-HOA) was analyzed at 8 mm. Corneal aberration measurement by Pentacam (Oculus) has been shown to be highly repeatable by McAlinden et al. [13]. The 3rd order corneal aberration coma has been suggested as grading parameter for KC in previous studies. For this study, we analyzed total 3rd order coma. Previous studies have included separate mentions of vertical and horizontal coma, and diagnostic roles of vertical coma. However, we felt that as the vertical and horizontal coma are Zernike decompositions of the total coma, there is a higher chance of a false outcome in terms of magnitude of change if these two parameters are studied independently over time. Using a combined product evaluated over time eliminates this potential source of error as the initial visit is automatically the ‘base scenario’.

Spherical aberration (SA) represents a predominantly central change in the corneal shape. Therefore, corneal anterior surface SA at 8 mm was included. As the net change in SA was more negative, directional bias in change was compensated by taking the sign inverse (-x) of rate readings for fractional change in SA when comparing to CA-HOA and coma (Additional file 1: Fig. S1). To avoid biases due to multiple comparison, no other individual Zernike modes were compared. Coma had a higher rate of fractional change compared to spherical aberration (*P* = 5.8 × 10^−4^, effect size = 0.3, Mann–Whitney test) and CA-HOA (*P* = 0.03, effect size = 0.2, Mann–Whitney test). Therefore, along with CA-HOA, which represents overall change in aberration profile, individual Zernike mode coma was included in further analysis.

#### Step 2: Intergroup analysis

The intra-group parameters with significantly higher fractional rate of change were compared. The pachymetric variables had a net negative direction for change (thinning). Therefore, to eliminate directional bias in the comparison of rate of change, the sign inverse (− x) values were taken for pachymetric variables when compared to the other variables. Coma had the largest rate of fractional change, followed by CA-HOA (*P* = 1.7 × 10^–14^, Kruskal–Wallis test). Both the aberrometric parameters (CA-HOA and anterior coma) had significantly higher rate of fractional change compared to all the other parameters (*P* values ranging from 1.4 × 10^−4^ to 7.4 × 10^−10^, Mann–Whitney test, effect size ranging from 0.4–0.7) (Table 2). The rate of fractional change was comparable between the pachymetric and the keratometric factors (*P* > 0.05 for all the comparisons, Mann–Whitney test) (Table 2).

Age and gender-based differences: The patient cohort was divided into two groups based on age cut-off of 30 years. There was no difference in fractional rate of change based on age and gender for any parameter (*P* > 0.05 in all the comparisons, Mann–Whitney test). There was no difference in the gender distribution for the two age groups (*P* > 0.05, Chi-square test).

## Discussion

In this study, we analyzed the progression rate of KC using pachymetric, keratometric and wavefront parameters on Scheimpflug imaging. Corneal wavefront parameters had significantly higher rate of progression compared to corneal thickness and keratometry. This is unlike what has been reported previously. Kosekahya et al. used Belin progression display from Scheimpflug imaging (Pentacam, Oculus, Wetzlar, Germany) to note that yearly change rates greater than 0.12 for anterior radii of curvature, 0.14 for posterior radius of curvature, 10.04 μm for thinnest pachymetry, and 0.68 D for Kmax, were the main factors indicating progression [14].

In most previous studies, central keratometry, Kmax or minimum corneal thickness were used as platform-independent measures to evaluate the progression of KC. These metrics are good for defining and monitoring the pathology at the cone’s apex. However, they describe a single point on the cornea and do not represent the peri-apical area of the cone. Changes in KC involve the entire area of the cone, and thus a worsening of the pathology can be missed if these single point estimations are used. A more comprehensive metric for the corneal distortion is the corneal wavefront as it summates the overall irregularity of the corneal surface for a given diameter into a single metric. Therefore, it is more intuitive to use corneal wavefront to assess the progression of ectasia. Our study demonstrated that in cases with progressive KC, worsening is noted faster in corneal aberrometry compared to other factors.

Additionally, front corneal surface coma and spherical aberration were compared. These two Zernike modes represent ectasia in different perspectives [15, 16]. The central component of progressive ectasia can be measured by the change (to more negative) for an already existing negative spherical aberration in KC. The paracentral component of progressive ectasia can be measured by coma and other asymmetric aberrations. Among the rotationally asymmetric aberrations, coma has been previously noted as an important predictive or classification factor for KC [16–19]. Even though almost all other individual Zernike modes, specially from the 3rd to 5th order, have been known to increase in KC, we refrained from using too many individual modes to prevent spurious correlation. We did see an increase in coma and spherical aberration (more negative for SA) in our study, however, the rate of change was statistically greater for coma. In fact, the rate of change for coma was more than that for CA-HOA. This can be explained by the fact that HOARMS is the root mean square summation of all higher-order aberrations, and the rate of change is dampened as a weighted average for the fractional change in all HOARMS. We would suggest that even though coma outperformed CA-HOA, both should be checked and monitored.

We did not use any proprietary or Pentacam specific classification systems to ensure that the findings of our study can be compared with other devices. There are other interesting observations in our study. The rate of change for Kmax and central keratometry were more than the pachymetric parameters. Among other factors, the Kmax is the most relevant keratometric factor used for the assessment of the status of the cone. However, centripetal extension of the cone (or worsening of the already central pathology in cases with centrally involving KC) can also be captured by the worsening of the central keratometry. Expectedly, the numerical change in Kmax was the highest, followed by the steep central and flat central keratometry. When adjusted for the fractional change over time, steep keratometry performed like Kmax. It is intuitive that over a period, the flattest part of the central cornea will be slowest to change, and this was confirmed in our study.

Among the pachymetric factors, rate of change was comparable in all three measured pachymetric indices. When screening for KC, minimum corneal thickness is an obvious pachymetric index of choice, as it ensures lower false negativity (and thus higher sensitivity) compared to other indices. However, the finding that the rate of fractional change was similar to other pachymetric indices gives a strong suggestion towards a more global change involving the thickness of the entire cone.

It was interesting to note that we did not find any difference in the rate of fractional change based on age. Our findings have a different scope of application compared to the accepted fact that the risk of progression becomes lesser with increasing age. As a selection criterion, we only looked at that cases that had progression, rather than looking at an overall incidence or rate of progression in a cohort of KC cases. We deduce that that if KC progresses, the rate of change can be independent of the age in the range we studied (16–45 years). Therefore, we do recommend a regular follow-up for cases more than 30 years of age, especially the ones at higher risk of progression.

The novel fractional rate of change index will help clinicians use a metric that is independent of time and baseline value. Our study shows that the index for corneal wavefront changes faster than keratometric and pachymetric values and therefore, if the index changes for wavefront values, it can be a useful indicator for clinicians of KC progression.

## Conclusions

The current study presents some novel findings in estimating the progression rate of KC. The main limitation of this study is a small sample size. The strengths of this study include a uniform cohort with robust data from a single center. If the end point values are higher over standardized time, this does mean that the parameter with higher values had a faster rate of change. As time is a linear construct, we must take two points and then work from it. With that understanding, we can deduce that the faster metric should have earlier discernable changes.

The fractional rate of change index will help clinicians use a time and baseline value independent metric. It is easy to calculate, and a simple excel/macro function can be created for the software of choice to calculate these values. This study shows that the index for corneal wavefront changes faster than keratometric and pachymetric values and therefore clinically, if the index changes for wavefront values, it can be a useful indicator for clinicians of KC progression.

### Supplementary Information

**Additional file 1: Figure S1. **Bar graph showing the values of the fractional rate of change for the parameters evaluated. Kmax, maximum keratometry (anterior); Ks, steep central keratometry (anterior); ACT, apical corneal thickness; CCT, central corneal thickness; TCT, thinnest central cornea; CV7, corneal volume at 7 mm; CV10, corneal volume at 10 mm; CA-HOA, corneal anterior surface higher-order aberrations at 8 mm; Coma, coma, anterior surface at 8 mm.

## Data Availability

The data presented in this study are available on request from the corresponding author.
