# Supplementary material for: Rate of fractional change in corneal tomography parameters in keratoconus using a combination of predictive parameters
Source: Eye Vis (Lond). 2023 Oct 1;10:40. doi: 10.1186/s40662-023-00357-y (PMC10544119; doi:10.1186/s40662-023-00357-y)

Figure S1: Bar graph showing the values of the fractional rate of change for the parameters evaluated. Kmax, maximum keratometry (anterior); Ks, steep central keratometry (anterior); ACT, apical corneal thickness; CCT, central corneal thickness; TCT, thinnest central cornea; CV7, corneal volume at 7 mm; CV10, corneal volume at 10 mm; CA-HOA, corneal anterior surface higher -order aberrations at 8 mm; Coma, coma, anterior surface at 8 mm


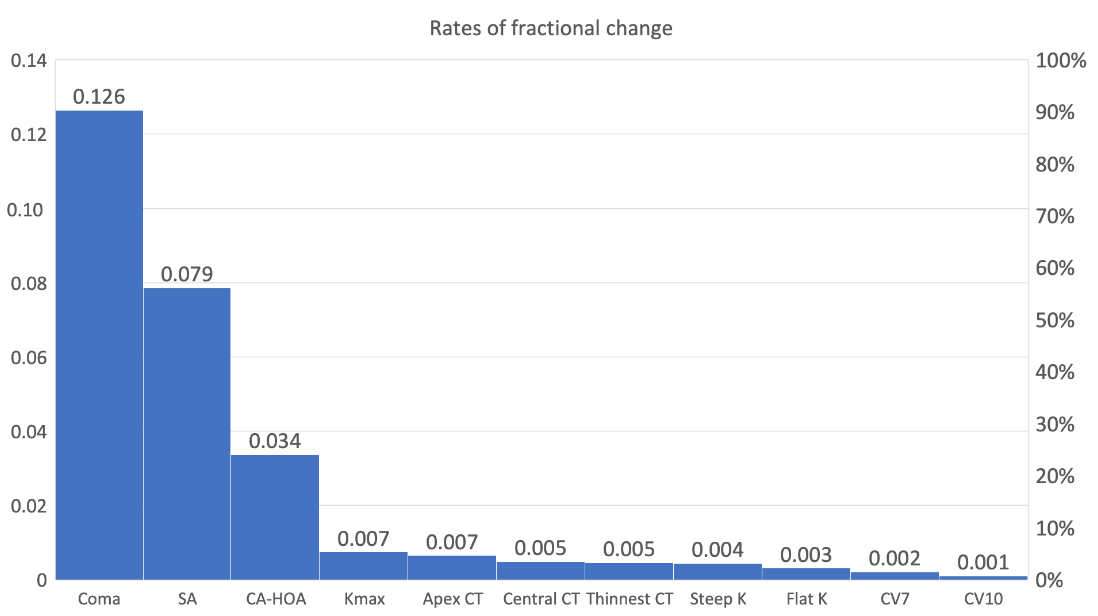

Supplement: Supplementary file 1 — Additional file 1: Figure S1. Bar graph showing the values of the fractional rate of change for the parameters evaluated. Kmax, maximum keratometry (anterior); Ks, steep central keratometry (anterior); ACT, apical corneal thickness; CCT, central corneal thickness; TCT, thinnest central cornea; CV7, corneal volume at 7 mm; CV10, corneal volume at 10 mm; CA-HOA, corneal anterior surface higher-order aberrations at 8 mm; Coma, coma, anterior surface at 8 mm. [file 40662_2023_357_MOESM1_ESM.docx]
